# Predictive effect of DCE-MRI and DWI in brain metastases from NSCLC

**DOI:** 10.1515/med-2021-0260

**Published:** 2021-08-26

**Authors:** Chengyu Ye, Quanbing Lin, Zhang Jin, Cuiping Zheng, Shenglin Ma

**Affiliations:** Department of Radiotherapy, Wenzhou Central Hospital, The Dingli Clinical Institute of Wenzhou Medical University, Wenzhou 325000, People’s Republic of China; Zhejiang Chinese Medical University, Hangzhou 310053, People’s Republic of China; Department of Radiotherapy, The First Affiliated Hospital, College of Medicine, Zhejiang University, No. 216, Huansha Road, Shangcheng District, Hangzhou 310006, Zhejiang Province, People’s Republic of China; Department of Haematology and Oncology, Wenzhou Central Hospital, The Dingli Clinical Institute of Wenzhou Medical University, No. 252, Eastern Baili Road, Lucheng District, Wenzhou 325000, Zhejiang Province, People’s Republic of China

**Keywords:** dynamic contrast-enhanced MRI, diffusion-weighted imaging, non-small cell lung cancer, brain metastasis, whole-brain radiotherapy, gefitinib

## Abstract

Non-small cell lung cancer (NSCLC), a commonly diagnosed lung cancer, is characterized by a high incidence of metastatic spread to the brain, which adversely impacts prognosis. The present study aimed to assess the value of combined dynamic contrast-enhanced MRI (DCE-MRI) and diffusion-weighted imaging (DWI) in predicting the treatment outcomes of whole-brain radiotherapy (WBRT) and gefitinib in brain metastases from non-small cell lung cancer (NSCLC) from the perspectives of response rate and short- and long-term efficacy. These results suggested that the indicators measured by DCE-MRI combined with DWI can be used as key imaging-derived markers that predicted the efficacy of WBRT combined with gefitinib in NSCLC patients with brain metastases. Specifically, patients with higher ΔADC_mid_ and ΔADC_post_ values showed better treatment outcomes. ROC curve analysis indicated ADC_post_, ΔADC_post_, ΔADC_post_ (%), and tumor regression rate as the best predictors of efficacy of WBRT combined with gefitinib in these patients. The short-term and long-term effects noted were also significant. Taken together, the findings of this study reveal that tumor regression rate, ADC_post_, ΔADC_post_, and ΔADC_post_ (%) can be used as important imaging indicators that predict the therapeutic effect of WBRT combined with gefitinib in NSCLC patients with brain metastases.

## 1 Introduction

The prevalence and mortality of lung cancer are alarmingly high across the globe [1]. Non-small cell lung cancer (NSCLC), accounting for about 80–85% of lung cancers, is commonly complicated by local and distal metastases, especially brain metastasis [2]. The high incidence of metastatic spread to the brain accounts for unsatisfactory prognosis of patients with NSCLC, and the median survival time of those untreated has been reported to be only about 1–3 months [3]. The currently utilized treatment strategies for brain metastasis from NSCLC chiefly include surgical treatment, radiotherapy, chemotherapy, targeted therapy, and immunotherapy [4]. However, fatal complications and a high possibility of recurrence frequently limit the efficacy of these treatment options, which calls for personalized medicine approaches to therapeutic strategies. Notably, gefitinib is a first-generation tyrosine kinase inhibitor (TKI) for NSCLC [5,6]. Emerging evidence supports whole-brain radiation therapy (WBRT) and epidermal growth factor tyrosine kinase inhibitors (EGFR-TKIs), such as gefitinib, erlotinib, and afatinib, for NSCLC patients affected by brain metastasis [7,8].

Dynamic contrast-enhanced MRI (DCE-MRI) and diffusion-weighted imaging (DWI) are commonly used methods to predict the treatment outcome of treatment methods in lung cancers [9]. DCE-MRI is understood to represent the pathological status of tumors by capturing angiogenesis and microvessel density characteristics, whereas therapeutic effects may be captured by DWI represented by alterations in apparent diffusion coefficient (ADC) values [10]. Magnetic resonance diffusion-weighted imaging is a special imaging method designed to detect diffusion of protons in water molecules within living tissues, where different *b*-value settings can facilitate the understanding of pathophysiological features as reflected by signal intensity. A study on the evaluation of changes after stereotactic body radiation therapy (SBRT) in NSCLC by DCE-MRI and DWI confirmed the feasibility of these two methods in evaluating the efficacy of NSCLC treatment [11]. Furthermore, WBRT and EGFR-TKI (gefitinib) are shown to have therapeutic effects on brain metastasis of NSCLC, and DCE-MRI and DWI can be used to evaluate the efficacy of this combined treatment. There is limited knowledge about the predictive value of DCE-MRI and DWI in evaluating the therapeutic effects of WBRT in combination with EGFR-TKI (gefitinib) on NSCLC patients with brain metastasis and was thus chosen as the focus of the present study.

## 2 Materials and methods

### 2.1 Study subjects

The study protocol was approved by the ethics committee of The First Affiliated Hospital, College of Medicine, Zhejiang University. All included patients provided signed informed consent. All study procedures were compliant with the Declaration of Helsinki. We collected 253 patients (202 males and 51 females; aged 42–85 years; average age of 67.00 ± 9.95 years) who were diagnosed with NSCLC with brain metastasis through histopathological clinical examinations at The First Affiliated Hospital, College of Medicine, Zhejiang University, from September 2010 to March 2015. The included subjects had a tumor diameter ≥2.0 cm and no contraindications for MRI. Clinical manifestations include symptoms related to lung cancer and the central nervous system, which mainly included headache, vomiting, epilepsy, convulsions, and limb dysfunction. Detailed patient inclusion criteria were as follows: (1) the diagnosis of lung cancer was confirmed by NSCLC histopathology or cytology; (2) Karnofsky performance status (KPS) score was above 50 and performance status (PS) score was not more than 2 before enrollment; (3) multiple treatments for the primary focus before enrollment were acceptable; (4) radiotherapy was acceptable after enrollment; (5) normal white blood cells, platelets, and liver and kidney functions; (6) EGFR exon 19 deletion or 21 mutation positive confirmed using polymerase chain reaction (PCR) analysis of peripheral blood samples, and expected survival time of >1 month. Exclusion criteria were as follows: (1) NSCLC patients with brain metastasis who have only diagnostic data based on head frequency enhanced scan; (2) patients with organ metastases other than lung and bone; (3) patients with previous acute cardiovascular events, such as myocardial infarction and stroke; (4) patients receiving surgery, radiotherapy, or gamma-knife treatment for intracranial metastases prior to enrollment; and (5) patients receiving targeted therapy before and after radiotherapy. All the included patients were treated with WBRT combined with gefitinib. Patients were divided into “sensitive” and “resistant” groups based on the WHO response evaluation criteria in solid tumors (RECIST, version 1.1) criteria [12]. MRI examinations (including MRI plain scan, MRI dynamic contrast-enhanced MRI (DCE-MRI) and diffusion-weighted imaging (DWI) scan) were performed on all patients before, during, and after treatment. Patients’ conditions were recorded before treatment on day 7, 14, 21, 28, and at 1 month after treatment. The ADC value and its change rate were calculated.

### 2.2 Therapeutic methods

All patients underwent whole-brain radiotherapy through 6 MV high-energy X-ray radiotherapy (2 Gray (Gy)/time, 5 times/week for 4 weeks, the total dose was 40 Gys), with the skull baseline as the lower boundary, the upper boundary, and the anterior and posterior boundaries open, with both sides opposite to the penetrating field. All patients were orally administered gefitinib (manufactured by AstraZeneca, UK) at 250 mg/day. Following radiotherapy, the patients continued to take medicine until they progressed, expired, or became drug intolerant due to drug toxicity.

### 2.3 Scanning methods

All patients were examined with a 3.0T magnetic resonance scanner (Discovery MR750 3.0T, GE, USA) with the HD8 channel head phased front ring, while in supine position with feet pointing toward the magnet (feet first supine). Plain MRI scans were performed before treatment, when the radiotherapy dose reached 20 Gy, and after treatment. Before treatment and after plain MRI scanning, dynamic enhanced and DWI sequence scanning were performed. DWI imaging was again performed once when the radiotherapy dose reached 20 Gy and once after the treatment was completed. The sequence and parameters of the three scans were kept consistent.

#### 2.3.1 Routine scanning (plain scanning)

After the positioning and scanning of the conventional horizontal axis, sagittal, and coronal plane, fast spin echo (FSE) sequence was used to scan the horizontal axis plane. T_1_ FLAIR (TR = 600–900 ms, TE = 5.8 ms, slice thickness = 7 mm, interval = 0.7 mm, matrix = 288 s × 192, NEX = 2, FOV = 38 cm), FRFSE T_2_WI (TR = 6,000–8,000 ms, TE = 85 ms, slice thickness = 6 mm, interval = l mm, matrix = 288 × 224, NEX = 2, FOV = 38 cm), and SE T_1_WI (TR 460 ms, TE 10 ms) were set, and the FSE T2 fat suppression sequence was controlled by respiratory gating.

#### 2.3.2 DCE-MRI scanning

Enhanced scanning was performed on patients with no contraindications. The sequence included 3D liver acquisition with volume acceleration (LAVA) dynamic enhanced scanning, and a 3D fast spoiled gradient-echo (FSPGR) sequence was applied to scan images of the whole pulmonary transverse plane and coronal plane in the delayed phase.

#### 2.3.3 DWI scanning

The scanning level was the same as the FSE T2 fat suppression sequence, and the breath trigger technology was used to allow the patients to breathe freely (respiratory training before scanning to ensure uniform breathing rhythm). DWI combined with ASSET technology and short-time flip to restore plane rotation were applied (slice thickness = 6 mm, interval = l mm, FOV = 38 cm, NEX = 4, matrix: 128 × 128, *b* = 0, 800 s/mm^2^).

### 2.4 Image processing and data measurement

All DCE-MRI and DWI images were transferred to the GEADW 4.6 workstation. Two expert radiologists jointly evaluated the image quality and reached a consensus after discussion. If the opinions were not uniform, another senior specialist was consulted to make consensus decisions. Image quality evaluation standards were as follows: (1) grade 1: good, the lesion is clear, and there is no obvious magnetic sensitive artifact; (2) grade 2: general, the lesion is slightly deformed, and part of the tumor structure is unclear; and (3) grade 3: poor, the lesion is seriously deformed and the tumor structure is unclear. The two examiners used the Functool software to measure the ADC value with DWI images of grades 1 and 2 blindly and manually sketched the maximum tumor layer as a region of interest (ROI). The areas with visible blood vessels, necrosis, obstructive atelectasis, and obstructive pneumonia were avoided. The specific steps were as follows: first, the plane with the maximum tumor size was selected, and the whole tumor region was taken as ROI to obtain the average ADC value of the tumor. Then, based on different colors of the ADC pseudo-color map, solid tumor areas with limited diffusion were identified, and ROIs were outlined as circles in areas with severe lesions and ellipses in areas with mild lesions to obtain the highest and lowest ADC values. The ROI area between the highest and lowest ADC values was kept at 30–50 mm^2^. In addition, images from plain and enhanced scans were observed to avoid areas of significant tumor necrosis and bleeding with high T_1_WI signaling. The mean ADC values of ADC_pre_, ADC_mid_, and ADC_post_ were each recorded. The rate of change of the mean ADC value before, during, and after treatment was calculated as follows: rate of change of average ADC value during treatment: ∆ADC_mid_ = (ADC_mid_ − ADC_pre_)/ADC_pre_ and rate of change of average ADC value at the end of treatment: ∆ADC_post_ = (ADC_post_ − ADC_pre_)/ADC_pre_.

#### 2.4.1 Calculation of tumor regression rate

All patients underwent brain DCE-MRI and DWI examination 1 month after the end of radiotherapy. The regression rate was calculated by combining the tumor diameter measured by DCE-MRI and DWI examination before treatment. The formula was as follows: tumor regression rate = (long diameter before treatment − long diameter 1 month after radiotherapy)/long diameter before treatment × 100% [13].

### 2.5 Efficacy evaluation criteria

Objective clinical efficacy was evaluated by measuring the tumor size, the change percentage of ADC value, and the short-term clinical efficacy of WBRT combined with gefitinib treatment. Based on RECIST standards, the efficacy was divided into four grades: complete response (CR): the tumor disappeared completely after treatment, partial response (PR): the maximum tumor diameter decreased by at least 30%, progressive disease (PD): maximum tumor diameter increased by 20% or more, and stable disease (SD): tumor changes between PR and PD. Patients with CR + PR or with SD + PD were allocated to the sensitive and resistant groups, respectively. In this experiment, the diameter of the tumor was measured by conventional MRI T_2_WI. The gold standard for the efficacy evaluation of the sensitive group was positive and that for the resistant group was negative. The receiver operating characteristic curve (ROC) was plotted, and the measured values of various parameters and their changes were calculated to determine the area under the curve (AUC). Once these results were obtained, the predictive values for therapeutic response at the optimal cut-off points were noted.

### 2.6 Follow-up

Patients underwent WBRT combined with gefitinib and three MRI scans. Data were collected from the patients regularly by telephone calls, follow-up visits, letters, or case reviews. The follow-up period lasted for 3 years or until their death. The follow-up rate was 100%. Through follow-up feedback, the changes in patient’s conditions, the short-term and long-term efficacy were recorded. The follow-up data, survival rate, and field recurrence rate were then analyzed.

### 2.7 Statistical analysis

All data were statistically analyzed using SPSS 21.0 software (SPSS Inc, Chicago, IL, USA). The descriptive data were summarized as the mean ± standard deviation. Independent sample *t*-tests were used for comparison between two groups, and one-way analysis of variance (ANOVA) and Tukey’s post-hoc tests were used for data comparison between multiple groups. Categorical data were summarized as percentages, and Chi-square test was applied. ROC curve analysis was used for prediction analysis. A value of *P* < 0.05 was considered as a statistically significant difference.

## 3 Results

### 3.1 Baseline characteristics

First, we analyzed and compared the differences in clinical characteristics between the two groups of NSCLC patients with brain metastasis, and general information for each group is listed in Table 1. There were no statistically significant differences in gender, average age, smoking history, primary pathological type, number of metastatic tumors, and maximum tumor diameter before treatment (all *P* > 0.05). Treatment outcomes of WBRT and gefitinib in NSCLC patients with brain metastasis were not significantly associated with gender, age, smoking history, primary pathological type, number of metastatic tumors, and tumor size before treatment.

**Table 1:** Clinical characteristics of 253 patients with NSCLC and brain metastasis

Characteristics	Sensitive group	Resistant group	*χ*^2^/*t*	*P*
**Sex**				
Male	107	95	0.562	0.454
Female	30	21		
**Age (years)**				
≤65	61	57	0.537	0.464
>65	76	59		
**Smoking history**				
Current and former smokers	100	86	0.042	0.837
Never smoking	37	30		
**Primary pathological type**				
Adenocarcinoma	56	45	2.649	0.449
Squamous carcinoma	37	38		
Adenosine carcinoma	29	17		
Magnocellular carcinoma	15	16		
**Number of metastases**				
≤3	96	84	0.168	0.682
>3	41	32		
Maximum tumor diameter before treatment (cm)	5.76 ± 0.58	5.70 ± 0.63	0.788	0.431

Note: *n* = 137 in the sensitive group and *n* = 116 in the resistant group. Enumeration data were expressed by cases, and a chi-square test was conducted. Measurement data were expressed by mean ± standard deviation. The significant difference between the sensitive group and the resistant group was compared by an independent sample *t*-test. A value of *P* < 0.05 was indicative of a significant statistical difference.

### 3.2 Image presentation

We could observe from conventional (plain scan) images and DCE-MRI that all the 253 patients included in the study presented multiple brain metastases from NSCLC, with a total of about 5,153 widely distributed lesions, ranging 2–7 cm in diameter, most of which were in the supratentorial tentorium. All lesions showed three enhanced forms on T_1_ DCE-MRI: there were 1,280 (24.84%) uniformly enhanced lesions with low signal on T_1_ FLAIR and high signal on T_2_WI, 3,438 (66.72%) of ring enhancement lesions with low or equal signal on T_1_ FLAIR and high or equal signal on T_2_WI, and 435 (8.44%) of liquid enhancement lesions with significantly low signal on T_1_ FLAIR and significantly high signal on T_2_WI. The ring walls were regular and uniform in thickness, with a thickness of 0.1–0.5 cm. Approximately 31.3% of the lesions showed varying degrees of edema around the lesions, with low signal on T_1_ FLAIR, high signal on T_2_WI, and equal signal on DWI. After DWI, based on grouping described earlier, about half of the uniformly enhanced lesions presented equal signals, while the rest presented high or low signals. There were high, low, equal, or mixed signals in the solid center of the ring enhancement lesions. Low signal was found in the fluid center of the ring enhancement lesions. High, low, equal, or mixed signals were found in percentages and tested with Chi-square tests. ROC curve was used for prediction analysis. A value of *P* < 0.05 was an indicative of significant statistical difference.

## 4 Results

### 4.1 Baseline characteristics

First, we analyzed and compared the differences in clinical characteristics between the two groups of NSCLC patients with brain metastasis. The general information of each group is listed in Table 1. There were no statistically significant differences in gender, average age, smoking history, primary pathological type, number of metastatic tumors, and maximum tumor diameter before treatment (all *P* > 0.05). Therefore, the treatment outcome of WBRT and gefitinib in NSCLC patients with brain metastasis was not significantly associated with gender, age, smoking history, primary pathological type, number of metastatic tumors, and tumor size before treatment.

### 4.2 Image presentation

**Figure 1:**
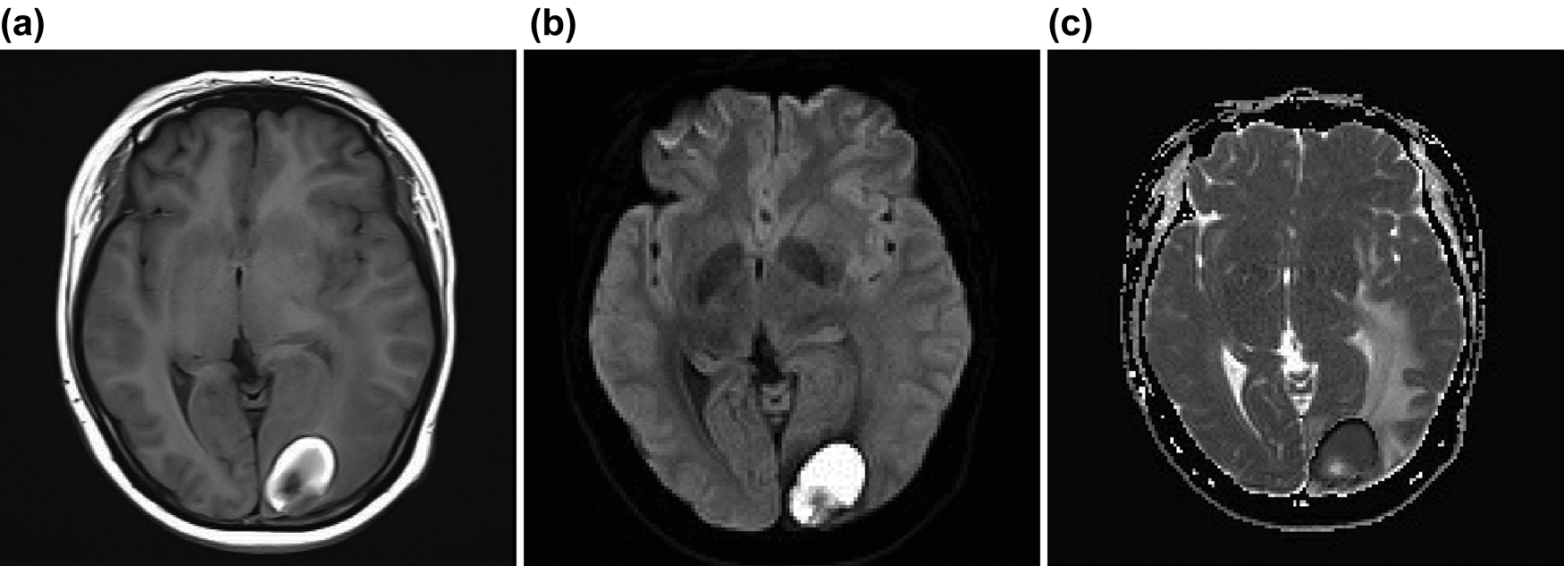
DCE-MRI, DWI, and ADC before treatment. Note: The depicted patient was male, 61 years old, with no smoking history, diagnosed with brain metastasis of squamous adenocarcinoma. (a) DCE-MRI image, showing a ring enhancement lesion about 4.3 cm in the right temporal lobe, with significantly low signal in the center (where the arrow points); (b) DWI image, showing significantly low signal in the center of the lesion, equal or high mixed signal at the edge, and equal signal in peritumor edema; (c) ADC image, showing accelerated diffusion of the lesion center and unrestricted diffusion of surrounding edema.

We could observe from conventional (plain scan) images and DCE-MRI that all the 253 patients included in the study presented multiple brain metastases from NSCLC, with a total of about 5,153 widely distributed lesions, 2–7 cm in diameter, most of which were supratentorial tentorium. All lesions had three enhanced forms on T_1_ DCE-MRI: there were about 1,280 (24.84%) of uniformly enhanced lesions with low signal on T_1_ FLAIR and high signal on T_2_WI, 3,438 (66.72%) of ring enhancement lesions with low or equal signal on T_1_ FLAIR and high or equal signal on T_2_WI, and 435 (8.44%) of liquid enhancement lesions with significantly low signal on T_1_ FLAIR and significantly high signal on T_2_WI. The ring walls were regular and uniform in thickness, with a thickness of 0.1–0.5 cm. Approximately 31.3% of the lesions have varying degrees of edema around the lesions, with low signal on T_1_ FLAIR, high signal on T_2_WI, and equal signal on DWI. After DWI, we observed that according to the grouping above, about half of the uniformly enhanced lesions presented equal signals, while the rest presented high or low signals. There were high, low, equal, or mixed signals in the solid center of the ring enhancement lesions. Low signal was found in the fluid center of the ring enhancement lesions. High, low, equal, or mixed signals were found in the wall of the lesions with ring enhancement. The edema around the lesion showed an equal signal. The image of a typical case in the sensitive group before treatment was selected as an example (Figure 1).

### 4.3 Average ADC value and its change rate at different time points

The average ADC value and its change rate in the sensitive and resistant groups during different treatment time points showed significant differences between the ADC_mid_, ADC_post_, ∆ADC_mid_, and ∆ADC_post_ values in the two groups (*P* < 0.05). Compared with the resistant group, the sensitive group showed higher ADC_mid_, ADC_post_, ∆ADC_mid,_ and ∆ADC_post_ (*P* < 0.05) values, as listed in Table 2. These results indicated that ADC value and its change rate were related to the efficacy of the combination therapy.

**Table 2:** Average ADC values and their rate of change at different time points (mean ± standard deviation)

	Sensitive group (*n* = 242)	Resistant group (*n* = 58)	*P*
**ADC value (10** ^**−3**^ ** mm** ^**2**^ **/s)**			
Prior treatment	1.01 ± 0.19	1.00 ± 0.20	0.843
Day 7 of treatment	1.13 ± 0.2^*^	1.14 ± 0.23^*^	0.814
Day 14 of treatment	1.35 ± 0.26^*#^	1.33 ± 0.27^*#^	0.524
Day 21 of treatment	1.68 ± 0.32^*#&^	1.60 ± 0.33^*#&^	0.073
Day 28 of treatment	2.13 ± 0.41^*#&$^	1.99 ± 0.41^*#&$@^	0.006
One month after radiotherapy	2.57 ± 0.50^*#&$△^	2.34 ± 0.49^*#&$△@^	<0.001
**ΔADC**			
Prior treatment–day 7	0.12 ± 0.02	0.13 ± 0.02^@^	<0.001
Days 7–14	0.20 ± 0.02^a^	0.17 ± 0.02^a@^	<0.001
Days 14–21	0.24 ± 0.03^b^	0.20 ± 0.02^b@^	<0.001
Days 21–28	0.27 ± 0.02^c^	0.24 ± 0.02^c@^	<0.001
Day 28–1 month after radiotherapy	0.21 ± 0.2^d^	0.18 ± 0.02^d@^	<0.001
Prior treatment–1 month after radiotherapy	1.55 ± 0.10^abcde^	1.33 ± 0.09^abcde@^	<0.001
**ΔADC (%)**			
Prior treatment–day 7	11.86 ± 1.55	13.11 ± 2.04^@^	<0.001
Days 7–14	19.91 ± 1.92^a^	17.31 ± 2.07^a@^	<0.001
Days 14–21	23.80 ± 2.91^b^	20.15 ± 1.96^b@^	<0.001
Days 21–28	26.95 ± 2.01^c^	23.97 ± 1.97^c@^	<0.001
Day 28–1 month after radiotherapy	20.98 ± 2.10^d^	17.92 ± 1.94^d@^	<0.001
Prior treatment–1 month after radiotherapy	155.03 ± 9.5^abcde^	133.04 ± 8.52^abcde@^	<0.001

Note: ^*^Compared with before treatment, *P* < 0.05; ^#^compared with day 7, *P* < 0.05; ^&^compared with day 14, *P* < 0.05; ^$^compared with day 21, *P* < 0.05; ^△^compared with day 28, *P* < 0.05; ^a^compared with prior treatment–day 7, *P* < 0.05; ^b^compared with day 7–day 14, *P* < 0.05; ^c^compared with day 14–day 21, *P* < 0.05; ^d^compared with day 21–day 28, *P* < 0.05; ^e^compared with day 28–1 month after radiotherapy, *P* < 0.05. ^**@**^Sensitive group compared with resistant group, *P* < 0.05.

### 4.4 Comparison of maximum tumor diameter and tumor regression rate at different time points

Differences in tumor maximum diameter and tumor regression rate between the two groups were compared, as listed in Table 3, there was no difference in the maximum tumor diameter between the two groups before treatment, on the 7th day and on the 14th day of treatment (*P* > 0.05). The maximum tumor diameter of both groups decreased at 21 days, 28 days after treatment, and 1 month after the completion of radiotherapy. The reduction in the resistant group was not statistically significant (*P* > 0.05), while that in the sensitive group was significantly reduced (*P* < 0.05). No significant difference was found in the change rate of maximum tumor diameter after the 7th day and the 14th day, ∆d_7_ (%) and ∆d_14_ (%) of the two groups (*P* > 0.05). Besides, a comparison of the change rate of maximum tumor diameter after the 21st day and the 28th day, ∆d_21_ (%) and ∆d_28_ (%), of the two groups showed no statistically significant difference (*P* > 0.05). One month after treatment, the maximum diameter change rate ∆d_1 month after radiotherapy_ (%) increased in both groups, and namely, the tumor regression rate increased, and the tumor regression rate was different between the two groups, and significantly increased in the sensitive group (*P* < 0.001). Moreover, the tumor regression rate in the sensitive group was significantly higher than that in the resistant group, and the maximum tumor diameter in the sensitive group was significantly lower than that in the resistant group (*P* < 0.001). These results indicated the good response of patients in the sensitive group to the combination therapy.

**Table 3:** Comparison of maximum tumor diameter and tumor regression rate at different time points (mean ± standard deviation)

Tumor maximum diameter (cm)	Sensitive group (*n* = 242)	Resistant group (*n* = 58)	*P*
Prior treatment	5.76 ± 0.58	5.70 ± 0.63	0.440
Day 7 of treatment	5.48 ± 0.57^*^	5.42 ± 0.60^*^	0.388
Day 14 of treatment	4.65 ± 0.48^*#^	5.61 ± 0.53^*#^	0.506
Day 21 of treatment	3.54 ± 0.42^*#&^	3.68 ± 0.45^*#&@^	0.011
Day 28 of treatment	2.12 ± 0.36^*#&$^	2.58 ± 0.33^*#&$@^	<0.001
One month after radiotherapy	1.16 ± 0.22^*#&$△^	1.66 ± 0.25^*#&$△@^	<0.001
∆d_7_ (%)	4.87 ± 1.31	4.98 ± 0.98	0.459
∆d_14_ (%)	15.17 ± 1.91^a^	14.88 ± 4.79	0.521
∆d_21_ (%)	23.79 ± 3.82^ab^	20.01 ± 2.91^@^	<0.001
∆d_28_ (%)	40.21 ± 7.28^abc^	30.01 ± 3.16^@^	<0.001
∆d_1 month after radiotherapy_ (%)	45.26 ± 4.08^abcs^	35.50 ± 4.63^@^	<0.001

Note: ^*^Compared with before treatment, *P* < 0.05; ^#^compared with day 7, *P* < 0.05; ^&^compared with day 14, *P* < 0.05; ^$^compared with day 21, *P* < 0.05; ^△^compared with day 28, *P* < 0.05; ^a^compared with prior treatment–day 7, *P* < 0.05; ^b^compared with day 7–day 14, *P* < 0.05; ^c^compared with day 14–day 21, *P* < 0.05; ^s^compared with day 21–day 28, *P* < 0.05. ^**@**^Sensitive group compared with resistant group, *P* < 0.05.

### 4.5 ROC curve to evaluate the efficacy of various radiographic indices in predicting the outcomes of brain metastases from NSCLC treated with WBRT and gefitinib

**Figure 2:**
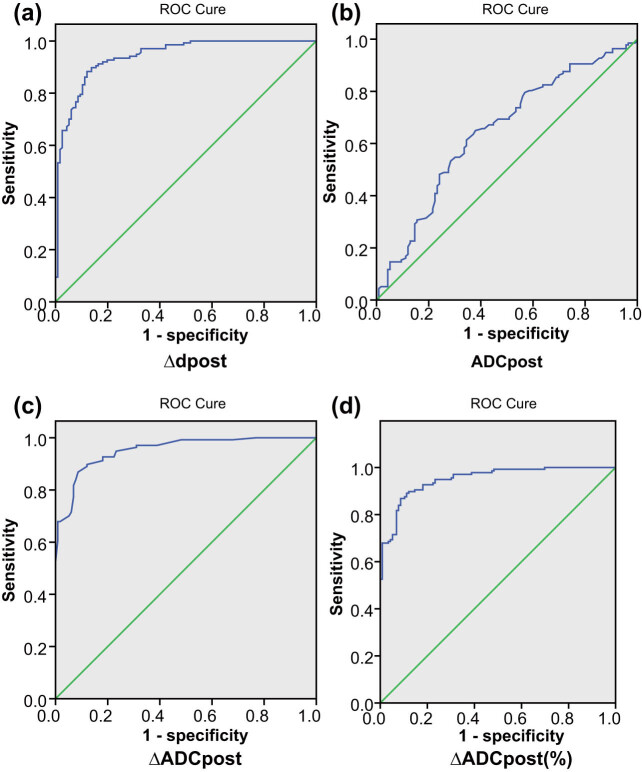
ROC curve analysis of ADC value. Note: (a) Δd_post_, (b) ADC_post_, (c) ΔADC_post_, and (d) ΔADC_post_ (%).

We plotted ROC curves based on tumor imaging indices obtained by DCE-MRI and DWI imaging. The ROC analysis results shown in Figure 2a revealed that sensitivity and specificity to predict the therapeutic effects of NSCLC were 88.3 and 87.9%, respectively, and the AUC was 0.941 when the tumor regression rate critical point was 40.64%. The critical point was taken as ADC_post_ value (2.46 × 10^−3^ mm^2^/s). The sensitivity and specificity for the prediction of therapeutic effect in NSCLC were 65.0 and 62.1%, respectively, and the AUC area was 0.645 (Figure 2b). After the treatment, the ROC curve (Figure 2c and d) indicated that the critical points were ΔADC_post_ = 1.46 and ΔADC_post_ (%) = 144.6%, with the sensitivity and specificity of prediction at 86.9 and 91.4, and 86.9 and 91.4%, respectively. The AUC areas were 0.953 and 0.951, respectively. These data indicated that the tumor regression rate, ADC_post_, ∆ADC_post_, and ∆ADC_post_ (%) were key imaging indicators for predicting the outcome of NSCLC patients with brain metastasis after WBRT and gefitinib treatment.

### 4.6 Short-term therapeutic effect

DCE-MRI and DWI examinations were performed in both groups after 3 months of radiotherapy. Based on the RECIST criteria, 253 patients were divided into two groups based on therapeutic effects. After 3 months of radiotherapy, the drug-sensitive group (*n* = 137) included patients with CR (*n* = 80) and PR (*n* = 57); the treatment-resistant group (*n* = 116) included patients with PD (*n* = 19) and SD (*n* = 97). Three months after treatment, the overall response rate was 54.15% (137/253) and the disease control rate was 45.85% (116/253).

### 4.7 Long-term prognosis

There were statistically significant differences in the KPS score, the number of survival improvement cases, and median survival time, i.e., 3-year survival rate after follow-up, between the two groups (*P* < 0.05), as listed in Table 4.

**Table 4:** Comparison of survival between the two groups

	Sensitive group (*n* = 137)	Resistant group (*n* = 116)	*χ* ^2^	*P*
**KPS score**				
Prior treatment	52.24 ± 4.57^*^	51.83 ± 4.72	0.700	0.484
Post treatment	70.31 ± 12.84^*^	59.18 ± 6.20	8.530	<0.001
Survival improvement cases (KPS ≥ 60)	98/137 (71.53%)^*^	53/116 (45.69%)	17.430	<0.001
Survival time (months)	27.70 ± 9.27^*^	20.66 ± 11.80	5.311	<0.001
Three-year survival rate	48.91% (67/137)^*^	33.62% (37/116)	7.506	0.006

Note: ^*^Sensitive group compared with a resistant group, *P* < 0.05.

## 5 Discussion

Lung cancer is the most frequently occurring cause of cancer-associated mortality worldwide, though the life expectancy of patients affected by lung cancer has been significantly prolonged with advances in systemic therapy [14]. In patients with NSCLC, metastatic spread to the brain significantly affects their prognosis and quality of life, as treatment efficacy is limited by the difficulty of most chemotherapeutic agents crossing the blood–brain barrier (BBB) [15,16]. WBRT, despite its complications, exerts a destructive effect on BBB, allowing more drugs to reach intracranial lesions [17]. When considering targeted drugs, previous evidence has indicated that gefitinib in combination with radiotherapy brought about significant improvement of quality of life in a Chinese population with brain metastases from NSCLC [18]. The current study provided evidence demonstrating that tumor regression rate, ADC_post_, ΔADC_post_, and ΔADC_post_ (%) can be used as important imaging indicators that predict the therapeutic effect of WBRT combined with gefitinib in NSCLC patients with brain metastases.

Prior evidence has identified EGFR gene mutations in NSCLC patients with brain metastasis [19]. Therefore, EGFR-TKIs, including gefitinib, plausibly offer significant remission of brain metastases of EGFR-mutated NSCLC [20]. Yang et al. found that gefitinib could augment the efficacy of WBRT in managing patients with brain metastases from NSCLC, as reflected by improved overall survival and progression-free survival [21]. However, treatment efficacy prediction tools for such combined treatment for brain metastases from NSCLC are lacking. In this study, DCE-MRI and DWI were used to observe and calculate various imaging parameters in NSCLC patients treated with WBRT combined with gefitinib, including mean ADC value and its change rate, tumor maximum diameter, and regression rate. By dynamically observing and comparing the tumor imaging of patients in the sensitive and resistant groups during different treatment periods, we determined that mean ADC value and its change rate and the tumor regression rate could sensitively and dynamically reflect the changes of tumor tissue occurring during treatment and the treatment effects, and this can be used as a valuable efficacy prediction tool.

At present, the major examination strategies for brain metastasis of NSCLC include computed tomography (CT), MRI, positron emission computed tomography/CT (PET-CT), serum tumor markers, lumbar puncture, and cerebrospinal fluid examination, and molecular pathological detection [22]. DCE-MRI and DWI have been found to enable differential diagnosis of benign and malignant soft tissue tumors, where quantitative analysis of tumors could refine diagnostic performance [23]. Studies have also focused on the diagnosis and differential diagnosis of DCE-MRI and/or DWI in NSCLC patients with brain metastases, showing these have good performance in the diagnosis of brain metastases from NSCLC [24,25]. The results of this study further demonstrated that indicators measured by DCE-MRI combined with DWI (tumor regression rate, ADC_post_, ΔADC_post_, and ΔADC_post_ (%)) can be used as imaging markers for predicting the efficacy of WBRT combined with gefitinib in NSCLC patients with brain metastases.

Earlier work has already shown that ADC can be used as a marker for a variety of cancers, assessing tumor grade, estimating progression-free survival, and predicting the early response of tumors to treatment [26]. In the current study, relative to the resistant individuals, the sensitive patients presented with higher ADC_mid_ and ADC_post_ and appreciably increased ΔADC_mid_ and ΔADC_post_. Furthermore, patients with higher ΔADC_mid_ and ΔADC_post_ values showed better treatment outcomes. These findings corroborate those of a previous study showing patients with brain metastases at 1 week and 1 month after treatment presented lower relative ADC value in radio responders than non-responders [27]. These data validate the utility of ADC to distinguish responders from non-responders as a biomarker for early radiation response.

The ROC curve analysis results suggested the utility of ADC_post_, ΔADC_post_, ΔADC_post_ (%), and tumor regression rate as the best predictors of WBRT combined with gefitinib in NSCLC patients with brain metastases. The short-term and long-term effects were also found significant. WBRT combined with gefitinib is well demonstrated in the treatment of NSCLC, and DCE-MRI and DWI can be used to evaluate its treatment efficacy in NSCLC patients with brain metastasis. Evidence also shows that in patients with brain metastasis, the use of radiotherapy followed by EGFR-TKI can improve prognosis and prolong overall survival time relative to EGFR-TKI treatment alone [28]. Accruing evidence indicates that gefitinib combined with radiotherapy is effective for the treatment of brain metastases from NSCLC; however, relatively small sample sizes could account for inconsistent results, and large-sampled clinical studies are essential.

In summary, this study revealed that tumor regression rate, ADC, ΔADC, and ΔADC (%) can be used as important imaging indicators to predict the therapeutic effect of WBRT combined with gefitinib in patients with brain metastases from NSCLC. The tumor response prediction tool established in this study mainly uses MRI technology to dynamically observe the physical characteristics of brain metastases, to establish the algorithm of ADC and regression rates. This approach is fundamentally different from biomarker prediction tools and is not limited by tumor type and treatment mode. In principle, the identified imaging markers could also be widely applied to a variety of brain tumors and brain metastases for outcome prediction of different treatment options. Thus, these could be of potential utility in a broad range of applications. Further research is also required to clarify the efficacy of these markers to predict outcomes of other treatments for brain metastases from NSCLC. Their predictive values for outcomes of other brain metastases or brain cancer also merit research to maximize the clinical translation of the identified imaging markers.
